# Impact of pharmaceutical care interventions on antidepressants adherence and clinical outcomes in depressed patients: A systematic review

**DOI:** 10.1016/j.rcsop.2025.100644

**Published:** 2025-08-20

**Authors:** Nirmal Raj Marasine, Sabina Sankhi, Shishir Paudel, Anisha Chalise, Rajendra Lamichhane

**Affiliations:** a Department of Pharmacy, CiST College, Baneshwor, Kathmandu, Nepal; b Department of Pharmacy, Shree College of Technology, Bharatpur, Chitwan, Nepal; c Kathmandu Institute of Child Health, Hepali Height, Kathmandu, Nepal; d Center for Research on Environment, Health and Population Activities (CREHPA), Lalitpur, Nepal; e School of Development and Social Engineering, Pokhara University, Kaski, Nepal

**Keywords:** Antidepressants, Clinical outcomes, Health related quality of life, Medication adherence, Pharmaceutical care interventions, Systematic review

## Abstract

**Background:**

Medication non-adherence, impaired health-related quality of life (HRQoL), increased depression severity, and patient dissatisfaction are common challenges among patients with depression. This systematic review aimed to evaluate the impact of pharmaceutical care interventions (PCIs) on antidepressant adherence, HRQoL, depression severity, and patient satisfaction.

**Methods:**

This review followed the Preferred Reporting Items for Systematic Reviews and Meta-Analyses (PRISMA) guidelines. A comprehensive search was conducted across PubMed, EMBASE, Web of Science, Scopus, PsycINFO, and CINAHL for randomized controlled trials (RCTs) published between 2000 and 2024. Studies evaluating pharmacist-led pharmaceutical care interventions aimed at improving antidepressant use and related outcomes were included. Data extraction and risk of bias assessment were performed using standardized forms and the Cochrane Collaboration's Risk of Bias tool.

**Results:**

Fifteen RCTs met the inclusion criteria. Common intervention strategies included patient education, counseling, telephone follow-ups, and drug monitoring. Nine studies reported statistically significant improvements in antidepressant adherence. Of the three studies assessing HRQoL, one demonstrated significant improvement. Four of thirteen studies showed a significant reduction in depression severity, and three of five studies reported increased patient satisfaction in the intervention group compared to controls**.**

**Conclusion:**

Pharmaceutical care interventions, particularly patient education and counseling, contribute meaningfully in improving antidepressant adherence and related patient outcomes. However, findings across studies are inconsistent due to variability in intervention components, measurement tools, delivery methods, and outcome measures. Further research should focus on well-designed, large randomized trials with standardized, therory-based interventions.

## 1 Introduction

Depression is a major public health concern, affecting over 300 million people globally and is a leading cause of disability among adults.^1^ Although antidepressants are clinically effective in managing depressive symptoms, real-world adherence remains low.^2^ Studies reported non-adherence rates to antidepressants ranging from 30 % to 97 %, which is notably higher compared to other psychotropic medications such as antipsychotics and mood stabilizers.^3^ Poor adherence contributes to treatment failure, relapse, progression to chronic depression, higher healthcare utilization, and diminished health-related quality of life (HRQoL).^4^^,^^5^

Multiple factors contribute to antidepressant non-adherence, including doubts about medication efficacy, fear of dependency, complex treatment regimens, side effects (e.g., sexual dysfunction), financial burden, inadequate patient–provider communication, and societal stigma surrounding mental illness.^6^, ^7^, ^8^ Studies have shown that treatment satisfaction is closely linked to medication adherence, particularly among individuals with chronic illnesses.^9^^,^^10^ Patients who perceive their treatment as effective and consistent with their expectations are more likely to adhere.^9^, ^10^, ^11^ Factors influencing treatment satisfaction include perceived effectiveness, side effect profile, convenience of dosage form, dosing frequency, and patients' positive beliefs about the treatment, all of which ultimately affect adherence.^11^

Pharmacists are among the most accessible healthcare professionals and can play a critical role in optimizing antidepressant use through direct patient engagement.^12^ In addition to dispensing medications, they increasingly contribute to patient care through services such as patient counseling, medication reviews, and therapeutic monitoring.^13^^,^^14^ Their frequent and trusted interactions with patients position them uniquely to address adherence barriers, manage side effects and drug interactions, provide targeted education, and alleviate concerns related to long-term use, medication costs, and pill burden.^14^ Pharmacist-led interventions may include medication counseling, adherence monitoring, side effect management, collaborative prescription adjustments, and ongoing patient support.^2^^,^^15^

Despite growing evidence supporting pharmacist-led interventions, few studies have specifically examined their impact on antidepressant adherence and associated clinical outcomes. Existing reviews often focus on broader psychiatric populations or general pharmaceutical care, with limited attention to depression-specific outcomes such as HRQoL and patient satisfaction.

This review provides a comprehensive synthesis of evidence on the effectiveness of pharmacist-led interventions in improving antidepressant adherence, HRQoL, patient satisfaction and depression severity in individuals with depression. The review is guided by three key research questions:

1. What types of pharmaceutical care interventions do pharmacists use to improve patient adherence to antidepressants and related clinical outcomes?
2. What impact do pharmaceutical care interventions have on patients' adherence to antidepressant medication?
3. How do pharmaceutical care interventions influence clinical outcomes, including HRQoL, depression severity, and patient satisfaction?

## 2 Methods

This systematic review was conducted in accordance with the Preferred Reporting Items for Systematic Reviews and Meta-Analyses (PRISMA) guidelines and was registered in the International Prospective Register of Systematic Reviews (PROSPERO) under the registration number CRD42021243231.

### 2.1 Search strategy

A systematic literature search was conducted to identify relevant studies examining the impact of pharmaceutical care interventions on antidepressant adherence and related clinical outcomes. The databases searched included PubMed, EMBASE, Web of Science, Scopus, PsycINFO, and CINAHL. The search covered studies published between January 2000 and January 2024 to capture contemporary pharmaceutical care interventions and reflect current clinical practices and antidepressant treatment guidelines. A combination of Medical Subject Headings (MeSH) and free-text keywords was used to construct a comprehensive search strategy. Primary search terms included: “depression,” “pharmaceutical care intervention,” “pharmacist intervention,” “medication adherence,” “health-related quality of life,” and “clinical outcomes”, were combined using Boolean operators (“OR” and “AND”) to identify eligible studies. Full search strings for each database are provided Appendix A. The search was last updated on January 15, 2024. Two independent authors (NRM and SS) screened all titles and abstracts for relevance and removed duplicates. Full-text articles of potentially eligible studies were then reviewed. Disagreements during screening or full-text review were resolved by consultation with a third reviewer (RL). Additionally, the reference lists of selected articles, systematic reviews, and meta-analyses were manually searched to identify any additional relevant studies. Out of 39 articles that met the initial screening criteria, 15 studies met the inclusion criteria and were included in the final systematic review.

### 2.2 Inclusion criteria

Studies were included in this review if they met the following criteria: (1) randomized controlled trials conducted in outpatient settings, including primary care clinics, community pharmacies, hospital outpatient departments, and telehealth services (2) studies involving patients aged 18 to 65 years diagnosed with depression and receiving antidepressant medications; (3) any type of pharmaceutical care interventions provided to patients to enhance medication adherence and clinical outcomes; (4) studies featuring a control or comparison group; (5) studies primarily focused on antidepressant adherence, including the measurement of adherence rates and/or clinical outcomes and patient satisfaction; and (6) full-text articles published in peer-reviewed journals between 2000 and 2024 in English. No restrictions were applied regarding the countries where the studies were conducted.

### 2.3 Exclusion criteria

The following studies were excluded: (1) non-randomized controlled trials, descriptive studies, pilot studies, editorials, book chapters, commentaries, qualitative interviews, systematic and narrative reviews, and studies conducted in inpatient settings; (2) studies involving patients younger than 18 years or older than 65 years, patients without depression, or those not prescribed antidepressants; (3) studies without the application of pharmaceutical care interventions for patients; (4) studies lacking a control or comparison group; (5) studies not primarily focused on antidepressant adherence or lacking measurements of adherence rates and/or clinical outcomes; and (6) studies published in non-peer-reviewed journals, those published before 2000, or those available in languages other than English.

### 2.4 Data extraction

The PICOSS (Populations, Interventions, Comparator, Outcomes, Setting and Study design) framework was used for data extraction. A standardized data extraction template was developed, and relevant data from all the eligible studies were extracted by SS and NRM. The data extraction template consisted of information on study details (author and year of publication), study objectives, study setting, follow-up, sample size, outcome measures, pharmaceutical care intervention details, and main findings of the studies.

### 2.5 Quality assessment

The Cochrane Collaboration's Risk of Bias Tool was used to assess the quality and risk of bias in each of the included randomized controlled trials.^16^ The assessment focused on various types of bias, including selection bias, performance bias, detection bias, attrition bias, reporting bias, and other potential sources. Each study was categorized as having a ‘high,’ ‘low,’ or ‘unclear’ risk of bias based on these criteria (Table 1). Two authors (SP and AC) independently performed the risk of bias assessment for each of the 15 studies selected for the comprehensive review. Although no studies were excluded based on their risk of bias, the quality assessments were taken into account during the interpretation of the results to provide context regarding the strength and reliability of the evidence. Any disagreements were resolved through discussion and consensus. Due to substantial heterogeneity in intervention components, outcome measures, settings, and follow-up durations, a meta-analysis was not conducted. Instead, a narrative synthesis was performed to integrate and summarize the findings of the included studies in a systematic manner.

**Table 1:** Risk of bias in randomized trials summary (*n* = 15).

Study (Author, Date) Source of Bias	Selection Bias		Performance Bias	Detection bias	Attrition bias	Reporting bias	Other bias	Score and Remark
	Random sequence generation	Allocation concealment	Blinding of participants & personnel	Blinding of outcome assessor	Incomplete outcome data	Selective reporting	Other source of bias	
Bultman and Svarstad, 2002^17^	**?**	**?**	**−**	**−**	**?**	**?**	**−**	Score-2: Technique used for systematic random sample selection is unclear, no blinding, unclear sample calculation
Finley et al., 2003^18^	**+**	**+**	**+**	**?**	**+**	**+**	**+**	Score-12: Unclear if assessors were blinded
Capoccia et al., 2004^19^	**?**	**?**	**−**	**−**	**+**	**+**	**+**	Score-8: Unclear randomization process, no blinding
Alder et al., 2004^20^	**+**	**+**	**?**	**?**	**+**	**+**	**+**	Score-10: No blinding of any personnel
Rickles et al., 2005^21^	**+**	**+**	**?**	**?**	**−**	**−**	**?**	Score-6: Unclear if any personnel were blinded, high loss to follow-up leading to potential selective outcome report
Al-Saffar et al., 2005^22^	**?**	**+**	**+**	**+**	**+**	**+**	**+**	Score-12: Unclear randomization process
Brook et al., 2005^23^	**?**	**+**	**?**	**?**	**+**	**+**	**+**	Score-8: Unclear randomization process and blinding
Crockett et al., 2006^24^	**?**	**?**	**?**	**?**	**−**	**+**	**−**	Score-4: Unclear of randomization and blinding information, 119 participants were recruited but 106 complete responses obtained resulting in risk of missing outcome data
Bosmans et al., 2007^25^	**?**	**+**	**?**	**?**	**+**	**+**	**+**	Score-8: Unclear randomization technique and blinding
Pyne et al., 2011^26^	**+**	**+**	**?**	**?**	**+**	**+**	**+**	Score-10: No information on blinding
Rubio-Valera et al., 2013^27^	**+**	**+**	**−**	**+**	**+**	**+**	**+**	Score-13: Reported blinding of participants and pharmacists was not possible
Marques et al., 2014^28^	**−**	**−**	**?**	**?**	**−**	**?**	**−**	Score-4: No information on blinding
Aljumah and Hassali, 2015^29^	**+**	**+**	**−**	**+**	**+**	**+**	**+**	Score-13: Pharmacists and psychiatrists were not blinded to the patients' group allocation
Cohen et al., 2020^30^	**+**	**+**	**?**	**?**	**+**	**+**	**+**	Score-10: No information on blinding
Yusuf et al., 2021^31^	**+**	**+**	**?**	**?**	**−**	**+**	**+**	Score-9: No information on blinding, not all 60 participants in each group provided complete data

Key: “+” low risk of bias = 2, “-” high risk of bias = 1, “?” unclear risk of bias = 0.

### 2.6 Outcome measures

The primary outcome assessed in this review was the impact of pharmaceutical care interventions on adherence to antidepressant medications. Secondary outcomes included the impact of these interventions on clinical parameters, specifically health-related quality of life (HRQoL), depression severity, and patient satisfaction.

## 3 Results

**Fig. 1:**
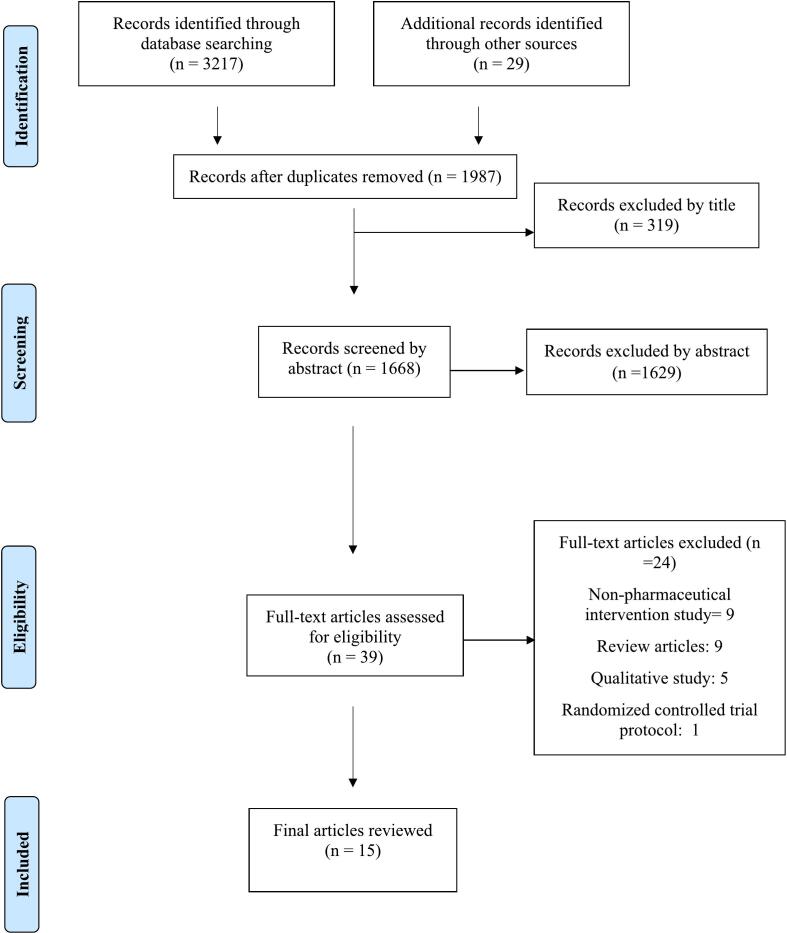
Article selection described by PRISMA flowchart.

A total of 3246 studies were identified through database searches. After removing 1259 duplicates, 1987 studies were screened by title and abstract independently by SS and NRM. Based on the eligibility criteria, 39 full-text studies were assessed, of which 15 met the inclusion criteria for this review (Fig. 1).

The included studies were randomized controlled trials (RCTs) published between 2000 and 2024, with sample sizes ranging from 60 to 533 participants. Among the 15 studies included in this review, the majority were conducted in the United States of America (7 studies), followed by Brazil (1), Kuwait (1), Netherlands (2), Nigeria (1), Saudi Arabia (1), Spain (1), and Australia (1).The duration of follow-up varied from 3 months to 2 years. The primary interventions involved patient education and counseling (12 studies), medication monitoring (9 studies), pharmacist-led collaborative care (6 studies), telephone follow-up (5 studies), and shared decision-making models (2 studies). The key findings of each study are summarized in detail (Table 2).

**Table 2:** : The description and key findings of the studies included in the systematic review (*n* = 15).

Author, year, country	Objective	Setting	Follow-up	n	Outcome measure	Pharmaceutical Care Interventions	Key findings
Bultman and Svarstad,^17^ 2002, USA	To assess the effects of pharmacist monitoring on patient satisfaction and adherence to antidepressant medication therapy.	Community pharmacies	2 months	100	Health Communication Model and Brief Medication Questionnaire for patient adherence.	Two telephone interviews assessing antidepressants history, beliefs, satisfaction, and adherence.	1. Within 3 months of the study, the medication adherence rate reached 76 %. 2.75 % of patients were satisfied with antidepressant treatment.
Finley et al.,^18^2003, USA	To determine the effects of a collaborative care model emphasized the role of clinical pharmacists in providing drug therapy management and treatment follow-up to patients with depression.	Primary care	6 months	125	Prescription refill records and follow-up frequency, Brief Inventory for Depressive Symptoms (BIDS).	Drug monitoring, patient education, and antidepressant drugs prescribing and titration under institutional protocol.	1. Medication adherence rose to 67 % in IG, 19 % more compared to CG (48 %). 2. Patients in collaborative care model were significantly more satisfied. 3. No significant changes in depressive symptoms.
Capoccia et al.,^19^ 2004, USA	To study the impact of pharmacist's interventions in collaborative care to improve the care of and outcomes for patients with depression.	Primary care	12 months	174	Patient self-report, Hopkins Symptom Checklist (SCL-20), Medical Outcomes Study Short Form 12 (SF-12)	Providing patient information, dosage adjustments, monitoring medication adherence, and adverse drug reaction management. Patients follow up by clinical pharmacists in conjugation with primary care providers and psychiatrists through telephone calls.	1. No statistically significant improvements observed in medication adherence, quality of life, depression symptoms and patient satisfaction between the groups.
Alder et al.,^20^ 2004, USA	To evaluate the outcome of pharmacist intervention in depressed primary care patients.	Primary care	3, and 6 months	533	Patient self-report, antidepressant use rate and severity of depression assessed by modified Beck Depression Inventory (BDI-II).	Each patient was contacted nine times over 18 months. Obtaining medication history, assessing drug related problems such as side effects or drug interaction, drug efficacy and toxicity for symptoms of depression, disease and drug education, and encouraging patients to maintain antidepressant therapy.	1. Overall, the antidepressant use rate increased by >11 % in the IG at six months (57.5 % vs. 46.2 %, *P* *<* *0*.03). 2. The intervention was significantly effective in improving antidepressant use among previously untreated patients in IG (32.3 % vs. 10.9 %, *P* < 0.001).
Rickles et al.,^21^2005, USA	To evaluate the effect of pharmacist led telemonitoring of antidepressant use on multiple outcomes of pharmacist–patient collaboration.	Community pharmacies	6 months	60	Self-report, frequency of patient feedback to pharmacist (FPFP), pharmacy and refill records, Beck Depression Inventory–II (BDI-II) score	Pharmacist guided education and monitoring (PGEM) and monthly telephone calls for three months. During the first call, patients were assessed with their antidepressant knowledge and beliefs, adverse effects, treatment goals and self-rate depression severity. During the second and third call, current adherence, new adverse effects and concerns and progress in medication goals were assessed.	1. PGEM significantly improved patient knowledge, medication beliefs, and perceptions. 2. PGEM patients missed fewer doses in 6 months compared to usual care (*P* < 0.05). 3. Depressive symptoms improved in both groups, but the difference was not statistically significant.
Al-Saffar et al., 2005,^22^ Kuwait	To assess the effect of information leaflets and counseling on Antidepressant adherence in depressed patients in Kuwait.	Hospital outpatients clinic	2, and 5 months	270	Patient self-report and tablet counting	Patients in treatment groups received patient information leaflets written in Arabic with counseling from a clinical pharmacist.	1. Providing a patient information leaflet (OR 3.0, CI 1.7–5.3) or leaflet plus counseling (OR 5.5, CI 3.2–9.6) significantly improved adherence. 2. Adherence rates increased from 10 % to 39 % at 2 months and 10 % to 43 % at 5 months in post-intervention.
Brook et al.,^23^2005, Netherlands	To enhance tricyclic antidepressant adherence among patients with depression through a pharmacist intervention.	Primary care	3, and 6 months	147	Electronic pill container (e-DEM), Hopkins Symptom Checklist Depression Subscale (SCL-13).	Provided with three coaching contacts that lasted 10–20 min, 25-min take-home video emphasizing importance of adherence and documentation of number of pills along with refill date.	1. No significant difference in adherence between groups in the intention-to-treat analysis (76 % vs 73 %). Per-protocol analysis showed a significant 17 % increase in adherence among intervention recipients (90 % vs 73 %). 3. No significant improvement in depressive symptoms observed.
Crockett et al.,^24^2006, Austrialia	Documentation and evaluation of patient outcomes in a pilot study into the role of rural community pharmacists in the management of depression.	Community pharmacies	1, and 2 months	106	Patient self-report, Kessler Psychological Distress Scale (K10)	Intervention pharmacists were provided with video-conference training on depression management and medication and were instructed to provide enhanced pharmacy service along with patient counseling and drug monitoring to the patients, while control group pharmacists were asked to provide usual care.	1. High adherence in both groups (96 % versus 95 %). 2. Both groups showed improvement in well-being, with a greater K10 score reduction in the intervention group (4.7 vs 4).
Bosmans et al.,^25^ 2007, Netherlands	To assess the cost-effectiveness of a pharmacy-based intervention to improve anti-depressant adherence in patients with depression.	Primary care	6 months	88	Electronic pill container (e-DEM) and Hopkins Symptom Checklist (SCL)	Patients were provided with three educational and coaching contacts focusing on the use of antidepressants. Also, offered take-home video marking the importance of medication adherence.	1. No significant difference observed in medication adherence with the intervention 2. Significant improvement in the Hopkins Symptom Checklist depression scores and treatment costs with the intervention.
Pyne et al.,^26^2011, USA	To assess the effectiveness of collaborative care for depression in HIV clinics.	Veterans Affairs HIV clinics	6, and 12 months	249	Hopkins Symptom Checklist (SCL-20), Psychological Health Questionnaire-9 (PHQ-9).	Patient education, assessment of depression symptoms and treatment monitoring, and instruction in self-management	1. Intervention group showed significantly higher treatment response (33.3 % vs 17.5 %) (OR: 2.50; 95 % CI: 1.37–4.56; *p* < 0.001). 2. Intervention participants experienced more depression free days during 12 months (β = 19.3; 95 % CI: 10.9–27.6; *p* < 0.001).
Rubio-Valera et al.,^27^2013,Spain	To evaluate the impact of a clinical pharmacist intervention on primary care patients who had initiated antidepressant treatment.	Primary care	3, and 6 months	179	Computerized pharmacy records, Psychological Health Questionnaire-9 (PHQ-9), EuroQol-5D (EQ-5D) and patient-satisfaction questionnaire	Patient education, medication information, and adherence.	1. Higher medication adherence in the intervention group at both 3 months (83.3 % vs 67.7 %) and 6 months (67.3 % vs 46.3 %) follow-up. 2.Significant improvement in HRQoL in the intervention group in both main (0.25 vs. 0.14) and per protocol analyses (0.27 vs. 0.16). 3. No statistically significant improvements observed in depression severity and patient satisfaction.
Marques et al.,^28^ 2014, Brazil	To study antidepressant drugs related problems, their negative clinical outcomes and the management of these problems through pharmacist interventions in outpatients with depressive disorder.	Clinic outpatient department	6 months	48	Beck Depression Inventory-II (BDI-II).	Education on disease, treatment, counseling on lifestyle habits, and pharmacotherapeutic follow-up.	After 6 months, 50 % of major depression cases were remitted and 44 % of moderate cases improved to mild depression.
Aljumah and Hassali.,^29^ 2015, Saudi Arabia	To assess whether the pharmacist intervention based on shared decision making (SDM) improved medication adherence and patient-related outcomes among depressed patients.	Hospital Pharmacy	6 months	239	Morisky Medication Adherence Scale (MMAS), Montgomery–Åsberg Depression Rating Scale (MADRS), EQ-5D-5L, and Treatment Satisfaction Questionnaire (TSQM 1.4).	Two Shared decision-making sessions on beliefs and knowledge about antidepressants.	1. The Intervention group showed significant improvements in medication adherence (+18 %) and treatment satisfaction (+6 %) at 6 months. 2. No significant differences between groups in changes in depression severity or HRQoL (*p* > 0.05).
Cohen et al.,^30^2020,USA	To assess whether a pharmacist-led telehealth disease management program is superior to usual nurse-led telehealth in improving diabetes and depression medication adherence in patients with concomitant diabetes and depression.	Tele-health	6-months	27	Pill counts and psychological health questionnaire-9 (PHQ-9) and Center for Epidemiologic Studies Depression Scale (CES-D).	In-depth medication review and telehealth sessions.	1. Significant improvement in antidepressant adherence (OR: 26; 95 % CI: 0.9–51.2; *p* < 0.05), and overall medication adherence (OR: 13.9; 95 % CI: 6.6–21.2; p < 0.05) in the pharmacist-led telehealth group at 6 months. 2.No significant improvement in overall depression scores.
Yusuf et al.,^31^2021, Nigeria	To assess the usefulness Sof pharmacist intervention to improve antidepressant medication adherence and disease severity in patients with major depressive disorder.	Psychiatric Hospital	3, and 6 months	120	Medication Adherence Rating Scale (MARS) and Beck Depression Inventory (BDI-II) instrument	Education, counseling, and follow-up contacts through mobile phones	1.Significant improvement in medication adherence (9.15 vs 5.22). 2. Decrease in disease severity (17.34 vs 21.40).

n = Sample size, IG: Intervention group; CG: Control group; OR: odd ratio; CI: Confidence interval.

### 3.1 Interventions

A wide range of pharmaceutical care interventions were implemented across the included studies to improve adherence to antidepressant therapy and enhance clinical outcomes. These interventions encompassed patient education and counseling, telephone interviews, structured follow-ups, medication monitoring, video or multimedia education, drug prescribing and dosage adjustments, collaborative care models, and shared decision-making approaches (Table 2).

Among these, patient education and counseling were the most commonly implemented strategies, reported in ten studies.^18^^,^^19^^,^^22^, ^23^, ^24^^,^^26^, ^27^, ^28^^,^^30^^,^^31^ These interventions focused on educating patients about antidepressant use, potential side effects, the importance of adherence, and reviewing their medication regimen. In addition, eight studies implemented telephone interview, education and follow-up^17^, ^18^, ^19^, ^20^, ^21^^,^^25^^,^^28^^,^^31^ to gather medication history, assess drug related concerns, monitor side effects and drug interactions, and reinforce adherence. Two studies utilized video or multimedia education as an adjunct to traditional counseling methods to further support patient understanding and engagement.^23^^,^^25^

Other pharmaceutical care interventions included drug monitoring to track adherence and detect adverse drug reactions^18^^,^^22^, ^23^, ^24^^,^^26^ as well as drug prescribing and dosage adjustments (titration) conducted under institutional protocols.^18^^,^^19^ Additionally, collaborative care models, where pharmacists worked as part of multidisciplinary teams to optimize pharmacotherapy, were implemented in two studies.^18^^,^^26^ Moreover, one study adopted a shared decision-making model, actively involving patients in treatment planning to enhance adherence and clinical outcomes.^29^

### 3.2 Adherence to antidepressants

Various approaches were employed to assess the impact of these interventions on antidepressant adherence (Table 2). Of the fifteen studies^17^, ^18^, ^19^, ^20^, ^21^, ^22^, ^23^, ^24^, ^25^, ^26^, ^27^, ^28^, ^29^, ^30^, ^31^ included in this review, thirteen specifically evaluated adherence outcomes.^17^, ^18^, ^19^, ^20^, ^21^, ^22^, ^23^, ^24^, ^25^^,^^27^^,^^29^, ^30^, ^31^ Among these, seven studies assessed adherence using patient self-reports and pharmacy records.^19^, ^20^, ^21^, ^22^^,^^24^^,^^27^ Additional methods included pill or tablet counting in two studies,^22^^,^^30^ electronic pill containers in two studies,^23^^,^^25^ the Morisky Medication Adherence Scale (MMAS) in one study,^29^ prescription refill tracking and follow-up frequency in one study,^18^ the Medication Adherence Rating Scale (MARS) in one study,^31^ and the Health Communication Model in one study.^17^

Nine studies^17^^,^^18^^,^^20^^,^^22^^,^^23^^,^^27^^,^^29^, ^30^, ^31^ reported statistically significant improvements in medication adherence following pharmaceutical care interventions. These studies implemented various strategies, including patient education and counseling,^22^^,^^23^^,^^27^^,^^30^^,^^31^ telephone interview and follow-up calls,^17^^,^^18^^,^^20^^,^^31^ drug monitoring,^18^^,^^22^^,^^23^ video or multimedia education,^23^ drug prescribing and dosage adjustment under institutional protocols,^18^ a collaborative care model,^18^ and a shared decision-making model.^29^ Conversely, four studies found no statistically ssignificant changes in adherence between the control and intervention group.^19^^,^^21^^,^^24^^,^^25^

### 3.3 Impact of intervention on patient clinical outcomes

The secondary outcomes assessed in this systematic review included the impact of pharmaceutical care interventions on HRQoL, depression severity, and patient satisfaction.

Depression severity was evaluated using a variety of validated tools: the Hopkins Symptom Checklist was employed in four studies,^19^^,^^23^^,^^25^^,^^26^ the Psychological Health Questionnaire-9 (PHQ-9) in three studies,^26^^,^^27^^,^^30^ and the Beck Depression Inventory (BDI-II) in four studies.^20^^,^^21^^,^^28^^,^^31^ Additional tools included the Brief Inventory for Depressive Symptoms (BIDS),^18^ the Montgomery–Asberg Depression Rating Scale (MADRS),^29^ and the Kessler Psychological Distress Scale (K10).^24^ HRQoL was assessed using the EuroQol-5D (EQ-5D) in two studies,^27^^,^^29^ and the Medical Outcomes Study Short Form 12 (SF-12) in one study.^19^ Two studies used the Treatment Satisfaction Questionnaire for Medication (TSQM)^27^^,^^29^ to evaluate treatment satisfaction, while one employed the Center for Epidemiologic Studies Depression Scale (CES-D).^30^

Among the three studies that evaluated HRQoL,^19^^,^^27^^,^^29^ only one^27^ reported a statistically significant improvement following patient information and counseling. The remaining two studies, which implemented patient education and counseling, telephone follow-ups, and dosage adjustments,^19^ and shared decision-making,^29^ did not observe statistically significant differences in HRQoL outcomes.

Of the thirteen studies that assessed the impact of pharmaceutical care interventions on depression severity,^18^, ^19^, ^20^, ^21^^,^^23^, ^24^, ^25^, ^26^, ^27^, ^28^, ^29^, ^30^, ^31^ four^25^^,^^26^^,^^28^^,^^31^ reported significant improvements in the intervention groups. These improvements were primarily associated with education and counseling,^26^^,^^28^^,^^31^ drug monitoring, collaborative care models,^26^ telephone interviews, education and follow up,^25^^,^^28^^,^^31^ and video and multimedia education.^25^ In contrast, nine studies^18^, ^19^, ^20^, ^21^^,^^23^^,^^24^^,^^27^^,^^29^^,^^30^ did not find statistically significant improvements in depression severity. Notably, one study^24^ found improvements in both intervention and control groups, making it difficult to attribute the changes solely to the intervention.

Five studies evaluated the impact of pharmaceutical care interventions on patient satisfaction.^17^, ^18^, ^19^^,^^27^^,^^29^ Of these, three^17^^,^^18^^,^^29^ reported significantly higher satisfaction levels in the intervention groups. Patients receiving telephone interviews, education and follow up,^17^^,^^18^ drug monitoring, drug prescribing and dosage adjustments, collaborative care,^18^ and shared decision-making interventions^29^ expressed greater satisfaction compared to those receiving usual care. However, two studies^19^^,^^27^ found no statistically significant difference in patient satisfaction between the intervention and control groups.

### 3.4 Quality assessment and risk of bias

The methodological quality of the 15 studies included in this review was assessed using the Cochrane Collaboration's risk of bias tool. Among these, five studies were rated as having a low risk of bias, six studies had an unclear risk, and four studies were categorized as having a high risk of bias, primarily due to incomplete blinding or high attrition rates (Table 2). For instance, the study by Bultman and Svarstad^17^ did not provide a clear description of the systematic random sampling technique or sample size calculation and lacked blinding procedures. Additionally, four studies did not clearly report details regarding blinding and randomization.^18^^,^^22^^,^^23^^,^^25^ Studies by Capoccia et al., Brook et al., and Bosmans et al., didn't implemented blinding,^19^^,^^23^^,^^25^ which may have influenced study outcomes. Similarly, there was no information on blinding of any personnel in the study conducted by Alder et al.,^20^ Pyne et al.,^26^ Marques et al.,^28^ and Cohen et al.,^31^ respectively. In a study by Rickles et al.,^21^ the absence of clear details on blinding, coupled with a high loss to follow-up, posed a potential risk of selective outcome reporting bias. Likewise, in Crockett et al.^24^ did not clearly describe their randomization and blinding methods, and of 119 participants recruited, only 106 provided complete response, leading to concerns about missing outcome data. The methodology reported by Yusuf et al.,^31^ did not specify blinding procedures, and complete data were not obtained from all the 60 participants. Additionally, the studies by Rubio-Valera et al.,^27^ and Aljumah and Hassali^29^ did not incorporate blinding for pharmacists and participants or, in the latter case, for pharmacists and psychiatrists.

## 4 Discussion

This systematic review provides a comprehensive evaluation of the impact of pharmaceutical care interventions on antidepressant adherence and related clinical outcomes. Data were extracted from fifteen randomized controlled trials conducted in different regions worldwide, assessing the role of pharmacist-led interventions in improving medication adherence, health-related quality of life (HRQoL), depression severity, and patient satisfaction.

Medication adherence is widely recognized as a key determinant of optimal treatment outcomes in mental health conditions, particularly depression.^32^ However, assessing the effectiveness of pharmacist-led interventions remains challenging due to heterogeneity in adherence measurement techniques across studies. Despite this methodological challenge, the present systematic review indicates that pharmaceutical care interventions significantly improve medication adherence among patients with depression. These findings underscore the indispensable role of pharmacists in designing and delivering targeted adherence-enhancing strategies within mental health care. Aligning with previous systematic reviews by Readdean et al.,^33^ Rubio-Valera et al.,^34^ and Bell et al.,^35^ this review further validates the positive impact of pharmacist interventions on adherence to antidepressant therapy. Notably, the improved adherence outcomes observed in this review may be attributed to the inclusion of five additional studies,^17^^,^^25^^,^^26^^,^^30^^,^^31^ not analyzed in the most recent systematic review by Readdean et al.,^33^ thereby enriching the current evidence base.

Depression, a leading psychological disorder, is known to significantly impair overall health, particularly the mental and social functioning domains of HRQoL.^36^ One study suggested that the statistically significant difference in HRQoL between the intervention and control groups might be partly influenced by the placebo effect.^27^ Nonetheless, pharmacist-led interventions that included patient education on the nature of disease, its prevention, and available treatment options likely contributed to positive changes in health beliefs, improved coping mechanisms, and reduced stigma, all of which may enhance patients perceived HRQoL.^27^ Notably, the EQ-5D instrument, employed in some studies to evaluate HRQoL, has recognized limitations, particularly in detecting subtle intergroup differences when baseline scores are comparable.^29^

While pharmaceutical care intervention has demonstrated a significant positive impact on antidepressant adherence, their effect on depression severity appears to be less conclusive. This highlights that improving medication adherence alone might not be sufficient to achieve an impactful reduction in depressive symptoms. Several factors beyond adherence, including pharmacodynamic response to antidepressants, diagnostic accuracy, patient-specific psychosocial variables, and environmental stressors may significantly influence clinical outcomes.^27^ Moreover, the presence of similar level of clinical improvement across both intervention and control groups in some studies indicates the complexity of treating depression, where multifaceted interventions may be required to achieve measurable symptom relief.^21^^,^^24^ These findings emphasize the need for a more integrated approach, combining pharmaceutical care with psychological, social, and medical support, to effectively manage depression and improve clinical outcomes.

A similar pattern of findings has been reported in previous systematic reviews by Readdean et al.,^33^ and Aljumah and Qureshi.^37^ Readdean et al.^33^ found that while eleven studies demonstrated significant improvements in medication adherence, only one showed a statistically significant reduction in depressive symptoms. Likewise, Aljumah and Qureshi^37^ observed that among eight studies reporting improved adherence to antidepressants, only two reported significant reductions in depressive symptoms.

In contrast, a systematic review by Ho et al.,^38^ identified a robust association between adherence and reduced depression severity. However, Ho et al.'s review predominantly included non-randomized clinical trials with follow-up periods exceeding six months, whereas most RCTs included in the present review had a follow-up duration limited to six months or less. This distinction suggests that the clinical benefits of pharmaceutical care interventions may require a longer-term implementation, potentially extending up to two years, to achieve measurable clinical improvements.^28^^,^^31^^,^^37^^,^^39^ Therefore, studies with extended follow-up periods may be better positioned to detect simultaneous improvements in both adherence and clinical outcomes.

Patient satisfaction has emerged as an important outcome in evaluating the effectiveness of pharmaceutical care interventions in mental health settings. In this review only five studies assessed patient satisfaction, of which three reported a statistically significant improvement among those receiving pharmacist-led interventions. These findings align with previous research^9^^,^^10^^,^^36^ and suggest that pharmacist involvement may enhance patient satisfaction when such outcomes are measured. Contributing factors may include improved pharmacist-patient communication, more accessible mental health support, and a better understanding of the illness and its management.^19^ Notably, studies reporting higher satisfaction also demonstrated improved adherence outcomes, reinforcing the positive relationship between patient satisfaction and medication adherence.^9^^,^^10^ These findings underscore the value of integrating patient-centered pharmaceutical care into mental health services to enhance both experiential and clinical outcomes.

This systematic review is among the few to comprehensively evaluate the effectiveness of pharmaceutical care interventions in enhancing adherence to antidepressants and related clinical outcomes. A key strength lies in its exclusive focus on randomized controlled trials (RCTs), the gold standard for assessing intervention effectiveness. Additionally, the inclusion of studies from diverse healthcare settings enhances the generalizability of the findings, offering a broader understanding of the impact of pharmacist-led interventions on medication adherence, depression severity, health-related quality of life (HRQoL), and patient satisfaction.

However, the review also has notable limitations. A major challenge is the substantial heterogeneity across studies in terms of intervention design, delivery, follow-up duration, adherence measures, and outcome definitions. This variability extends beyond methodological issues, reflecting a broader lack of standardization in how pharmaceutical care interventions are conceptualized and delivered. For instance, while patient education and counseling were commonly used, their effectiveness varied, highlighting the need to better define content, quality, and delivery methods. Due to this heterogeneity, a meta-analysis was not feasible, limiting pooled effect estimation and direct comparisons. Furthermore, limiting the search to English-language studies may have excluded relevant research, introducing potential language and publication bias.

To advance this field, future studies should adopt theory-based, well-defined intervention frameworks that specify core components, intensity, and delivery methods. Standardized outcomes, adequate follow-up, and process evaluations are essential to understand not just whether interventions work, but how and why. Tailoring interventions to individual needs will support the development of more effective and scalable pharmacist-led strategies for managing depression.

## 5 Conclusion

This systematic review highlights the significant contribution of pharmacist-led pharmaceutical care interventions in improving adherence to antidepressant medications. Interventions incorporating patient education and counseling, structured telephone follow-ups, drug therapy monitoring, and collaborative care models consistently improved adherence outcomes. However, evidence on their impact on broader clinical outcomes, such as depression severity and health-related quality of life (HRQoL) remains limited and inconsistent. Future research should focus on well-designed, larger randomized trials with standardized interventions and consistent outcome measures. Strengthening continuity of care, tailoring interventions to individual needs, and addressing psycho-social factors may further optimize the effectiveness of pharmaceutical care in managing depression.

## Declaration of use of generative AI in writing

The authors declared that no generative AI tools were used in the preparation of this manuscript.

## CRediT authorship contribution statement

**Nirmal Raj Marasine:** Writing – review & editing, Writing – original draft, Visualization, Validation, Supervision, Methodology, Investigation, Formal analysis, Data curation, Conceptualization. **Sabina Sankhi:** Writing – review & editing, Writing – original draft, Methodology, Formal analysis, Data curation, Conceptualization. **Shishir Paudel:** Writing – review & editing, Methodology. **Anisha Chalise:** Writing – review & editing. **Rajendra Lamichhane:** Writing – review & editing.

## Funding

None.

## Declaration of competing interest

The authors declare that they have no known competing financial interests or personal relationships that could have appeared to influence the work reported in this paper.

## Appendix A Full search strings for each database

**PubMed**

(“Depression”[Mesh] OR depression[tiab]) AND

(“Pharmaceutical Services”[Mesh] OR “pharmaceutical care”[tiab] OR “pharmacist intervention”[tiab]) AND

(“Medication Adherence”[Mesh] OR “medication adherence”[tiab]) AND

(“Quality of Life”[Mesh] OR “health-related quality of life”[tiab] OR HRQoL[tiab]) AND

(“Treatment Outcome”[Mesh] OR “clinical outcomes”[tiab])

• [Mesh] = Medical Subject Headings (controlled vocabulary).
• [tiab] = Title and Abstract fields (free-text search).

**EMBASE**

(‘depression’/exp. OR depression: ti,ab) AND

(‘pharmaceutical care’/exp. OR ‘pharmacist intervention’:ti,ab) AND

(‘medication adherence’/exp. OR ‘medication adherence’:ti,ab) AND

(‘quality of life’/exp. OR ‘health-related quality of life’:ti,ab OR HRQoL: ti,ab) AND

(‘clinical outcome’/exp. OR ‘clinical outcomes’:ti,ab)

• /exp. = Exploded Emtree term (includes the term and its narrower terms).
• :ti,ab = Title and Abstract fields (free-text search).

**Web of Science**

TS = (depression AND (“pharmaceutical care” OR “pharmacist intervention”) AND

“medication adherence” AND (“health-related quality of life” OR HRQoL) AND “clinical outcomes”).

• TS= = Topic search (includes title, abstract, author keywords, and Keywords Plus).

**Scopus**

TITLE-ABS-KEY(depression AND (“pharmaceutical care” OR “pharmacist intervention”) AND

“medication adherence” AND (“health-related quality of life” OR HRQoL) AND “clinical outcomes”).

• TITLE-ABS-KEY = Searches in title, abstract, and keywords.

**PsycINFO**

(depression AND (“pharmaceutical care” OR “pharmacist intervention”) AND

“medication adherence” AND (“health-related quality of life” OR HRQoL) AND “clinical outcomes”).ti,ab.

• .ti,ab = Restricts the search to title and abstract.

**CINAHL**

(depression AND (“pharmaceutical care” OR “pharmacist intervention”) AND

“medication adherence” AND (“health-related quality of life” OR HRQoL) AND “clinical outcomes”)
